# A Proposed Molecular Mechanism for Physical Analgesia in Chronic Pain

**DOI:** 10.1155/2018/1260285

**Published:** 2018-05-20

**Authors:** Norio Saito, Rei Shima, Yurika Yamada, Masaru Nagaoka, Etsuro Ito, Tohru Yoshioka

**Affiliations:** ^1^Research Institute for Elderly Health, Waseda University, 2-579-15 Mikashima, Tokorozawa, Saitama 359-1192, Japan; ^2^Department of Biology, Waseda University, 2-2 Wakamatsucho, Shinjuku, Tokyo 162-8480, Japan; ^3^Graduate Institute of Medicine, Kaohsiung Medical University, 100 Shih-Chuan 1st Road, Sanmin, Kaohsiung 80708, Taiwan

## Abstract

Although pain is indispensable for survival, chronic pain places a heavy burden on humans. As the efficacy of opioid treatment is limited, the development of alternative methods of pain relief without medication is desirable. Recently, we have developed a novel method of physical analgesia using an adhesive “pyramidal thorn patch.” When we apply about 3 trials of these patches on the skin of a pain region, the pain region moves toward the spinal cord like a “cutaneous rabbit,” and finally, the pain vanishes. In the present review, we propose a molecular mechanism for this analgesic method or pain relief following application of the pyramidal thorn patch where firstly the mechanoreceptors and their related nerves under the skin are activated in response to touch. Transient receptor potential (TRP) channels serve as mechanosensitive channels within these mechanoreceptors. We further propose that activation of the nerves connected with the mechanoreceptors releases oxytocin, which has an antinociceptive function and activates TRP channels to hyperpolarize the pain signal nerves. We believe that our system will pave the way for alternative pain treatment.

## 1. Introduction

In 2006, Cox et al. identified SCN9A, the gene encoding a voltage-dependent Na^+^ channel expressed in nociceptive neurons, and found that it is strongly related to pain sensation in three northern Pakistan families investigated in their study [1]. Family members inherited a mutation in this gene that left them with no sense of pain, and they consequently died at an early age. Kremeyer and his coworkers later linked the transient receptor potential cation channel, subfamily A member 1 (TRPA1 channel), which is activated by cold temperature and is permeable to Na^+^, to a pain syndrome [2]. Both of these reports afforded an opportunity to reconsider the nature of pain manifestation and the neurological mechanism behind pain sensation.

Pain sensation is considered indispensable for survival, leading to the protective behavior necessary for injured tissues to heal. Indeed, pain is associated not only with damaged tissue but also frequently with serious illnesses [3, 4]. For a long time, the senses of pain and itch have been thought as a similar sense: “itch is a low-level form of pain.” However, recently, this idea was totally changed, because the pain sensation and the itch one are recognized as different senses in the prefrontal cortex [5]. Thus, pain must be studied independently.

The molecular mechanisms underlying pain sensation are poorly understood at present. A significant barrier to elucidation is the paucity of experimental models. Adequate modeling of a pain system *in vitro*, for example, would require some combination of keratinocytes and nerve endings, cultures of neurons and glial cells in the dorsal root ganglion (DRG) and the dorsal horn (DH), or other neuronal networks of the brain. Existing textbook models of pain sensation posit a hypothesis based on a combination of macroscopic evidence of nerve anatomy and insufficient evidence of neurophysiology [6]. A particular problem is the role of DH in pain relief: outside of medicine, this is deadlocked by lack of the knowledge of molecular mechanisms involved [7]. The most serious concern, perhaps, is that there is currently no instrument available to clinics that can quantitatively measure the degree of patient pain (https://www.rch.org.au/rchcpg/hospital_clinical_guideline_index/Pain_Assessment_and_Measurement/).

According to the definition by the International Association for the Study of Pain, “pain is an unpleasant sensory and emotional experience associated with actual or potential tissue damage or described in terms of such damage” (https://www.iasp-pain.org/Taxonomy). At present, there are 3 categories of pain distinguishable by source: nociceptive pain, inflammatory pain, and pathological pain [8, 9]. Furthermore, based on the duration of sensation, pain can be divided into another 3 types: transient pain, acute pain, and chronic pain [10]. Transient pain is induced by the activation of nociceptive receptors, mainly in the skin, with little or no tissue damage, whereas acute pain is associated with local tissue damage [11]. Chronic pain, which arises from damage to the internal or subcutaneous organs, is not easily relieved, largely because the affected organs and their associated peripheral nerves are difficult to be entirely cured by medicine without side effects. In the case of phantom limb pain, this cannot be relieved because it is impossible for the lost nerve to regenerate [12, 13].

The methods we are about to propose in this review may lead to the relief of pain through the application of physical stimulation (mechanical pressure, vibration, electric current, etc.), as demonstrated by the example of knee arthritis [14]. Up to now, pain reduction by physical stimulation treatment has been categorized outside the field of medicine, as an “alternative medicine” or “modality.” The proposed treatment is based on the concept of a “soft touch in a relaxed atmosphere,” which can promote a mechanoreceptor-stimulated effect as well as a psychological effect, whose mechanisms are still essentially unknown [15, 16]. Of particular interest here is the phenomenon of phantom limb pain, which is associated with neither a damaged tissue area nor an inflamed part of the body, and is therefore thought to be a placebo effect evoked inside the brain [17]. If, indeed, this is a result of a phenomenon termed “proprioceptive memory” as Weeks et al. have proposed [18], then mindfulness meditation could also constitute an influential candidate as another type of pain treatment [19].

**Figure 1:**
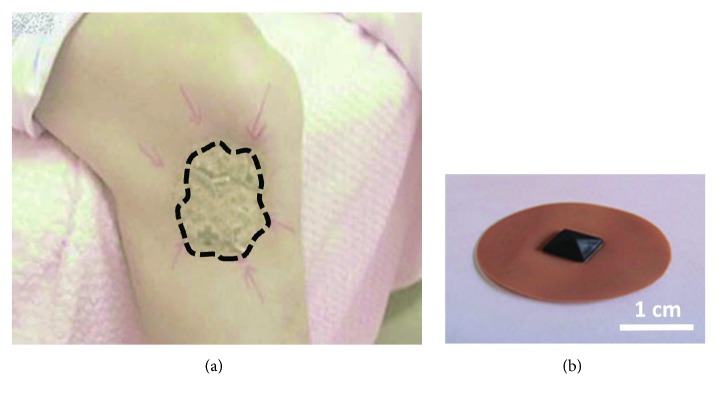
Pyramidal thorn patch treatment. (a) Several pyramidal thorn patches are applied at the pain region. The pain region moves like a “cutaneous rabbit” toward the spinal cord. (b) Patch with silicon pyramidal thorn. Diameter is 3 cm.

We have recently developed a novel physical analgesia (Figure 1). The technology uses a circular adhesive patch, 3 cm in diameter, with a silicone spine in the form of a pyramidal thorn (Figure 1(b)). We temporally call this patch a “pyramidal thorn patch.” When applied to the elbow, shoulder or knee, chronic pain is promptly relieved after ca. 3 trials of application [20]. Interestingly, application of pyramidal thorn patches mobilizes the pain region like a “cutaneous rabbit” [21], and finally, the pain sensation was completely dispelled. A so-called “cutaneous rabbit” is the phenomenon that rapid sequential taps delivered first to one location and then to another on the skin create the somatosensory illusion that the tapping is occurring at intermediate locations between the actual stimulus sites, as if a small rabbit was hopping along the skin from the first site to the second [21]. This patch-applied pain relief is “soft touch-induced analgesia.” Patches are applied directly to the pain region so that both the nerve signals from the patched area and those from the pain area target the same DRG and DH. From this data, we can propose a molecular model for physical analgesia that leads to pain signal reduction by a glia-neuron interaction in the DRG and DH [22].

## 2. Mechanoreceptors and Associated Nerves in the Skin

The epidermal mechanoreceptors for touch are composed of Meissner corpuscles, Merkel cells, and free nerve endings. In the dermis, there are Ruffini endings and Pacinian corpuscles in a glabrous (or hairless) skin, whereas there is a complex combination of mechanoreceptors and their associated nerves and hair follicle receptors in a hairy skin [23, 24]. Lumpkin and Caterina initially proposed that Merkel cells, nerve endings, and the guards of hair follicles are connected to A*β* and C fibers [23]. Later, this model was revised when low-threshold mechanoreceptors (LTMR) were found to be the major activators of A*β* and C fibers, and LTMR around hair follicles were posited to activate C fibers. These mechanoreceptors are selectively connected to the nerves. Meissner corpuscles connect to A*α* and *β* fibers for stroking, Merkel cells connect to A*α* and *β* fibers for pressure, and Ruffini corpuscles connect to A*α* and *β* fibers during skin stretching. The primary sensory afferents innervating the human skin are connected to the nerves as follows: discriminative touch nociceptors for pain are connected to A*β* fibers, nociceptors for pain connect to A*δ* fibers, and polymodal nociceptors for pain, emotional touch, and itch connect to C fibers [25].

Ordinarily, contact with the skin surface activates Ruffini corpuscle endings and Merkel cell-neurite complexes, with the Merkel cells producing long-lasting nerve impulses. Both types of receptors may be the main pain receptors for tissue damage and inflammation [26, 27]. Soft touches are known to be important for pain relief [16]. The application of pyramidal thorn patches recapitulates soft touches, which are sensed in up to three ways: free nerve endings, lanceolate endings around the hair follicle, and probably Meissner corpuscles in the glabrous skin as well. Strong touches, on the other hand, activate two, deeper receptors: the Pacinian and Ruffini endings. Both of these nerve endings are sensitive to strong pressure and vibration. Thus, soft touches and vibration have the potential for developing a novel type of analgesia, similar to that used to treat knee arthritis as mentioned above [23]. We propose a number of different experimental models using various combinations of mechanoreceptors and nerves (Table 1). Our pyramidal thorn patches may activate C fiber-LTMR, in particular, A*δ* fiber-LTMR, A*β* fiber-rapidly adapting (RA1), and the Merkel cell-A*α*/*β* fibers that are activated by soft touches [28]. All these mechanoreceptors were found to have pressure-sensitive molecules, such as mechanosensitive TRP channels, on their cell surface [29]. The electric properties of these TRP channels have been physiologically measured, and their ionic permeability deduced (i.e., for Meissner corpuscles, Merkel cells, and free nerve endings) according to their firing patterns [30, 31].

Currently, the most important issue to be resolved in analgesia is how soft touches induce pain relief at the molecular level. To this end, it is first necessary to determine the primary localization of analgesia and/or reduction of pain between the central nervous system (CNS) and the peripheral nervous system (PNS). In the case of pharmacological analgesia, where an afferent pain signal travels to the brain, opioid treatment is thought to augment the 5-HTergic signal descending from the brain to the DH [25]. However, this type of model fails to adequately explain why soft touches can relieve pain. To answer that question, an “oxytocin hypothesis” in the DRG and DH will be posited later, in Section 5.1.

## 3. Role of TRP Channels in Mechanosensitivity of the Cell Membrane

According to the cell biology theory of pain, the initial event of pain sensation in the skin occurs at free nerve endings, namely, lanceolate endings and Meissner cells [25]. Lanceolate endings produce pulse trains during hair flattening, and Meissner cells produce long-lasting but irregular pulse trains if the touch is feather-light (group I). Merkel cells and Ruffini endings show slowly adapting irregular pulse trains (group II). Pacinian corpuscles and a part of the lanceolate endings surrounding hair roots sometimes show rapidly adapting firing responses. From this, we posit that to achieve a long-lasting effect, vibration may be the most potent type of stimulation (Table 1).

These mechanoreceptors have various types of mechanosensitive (TRP) channels: TRPV1–V4, TRPA1, TRPM8, and TRPCs in keratinocytes [23]. According to our hypothesis, the soft touch receptors must be located just under the glabrous skin surface or around the hair follicles in the hairy skin, as in Merkel cells and nerve endings generating sparse firings. The normal (or usual) touch receptors must not be very sensitive to soft touches and, moreover, generate long-lasting dense firings. The last types are Pacinian corpuscles, deeply located with fast-adapting characteristics (a single pulse). To produce these 3 types of firing, each mechanoreceptor has to have different types of TRP channel receptors, which can produce different values of ionic selectivity for Ca^+^ and Na^+^ for the same values of conductance [32].

### 3.1. TRP Channels in Transient Touch-Sensing Cells of the Hairy Skin

What is the essential characteristic of TRP channels that controls the duration of depolarization? If we assume that the opening duration of each TRP channel is the same, membrane potential change must be controlled by a reversal of potential and determined by the permeability ratio of Ca^2+^ and Na^+^ (PCa/PNa). This permeability ratio, a function of reversal of potential, can determine firing frequency [32]. The firing caused by the soft mechanical touches in free nerve endings and lanceolate endings probably activates TRPA1, TRPC3 and 4, TRPV2, and TRPML1. The permeability ratio (PCa/PNa) of these TRP channels is about 1 : 2. These channels have a conductance of about 50 pS on average, and the reversal potential is theoretically estimated to be 5–10 mV. The Merkel cell-neurite complex is also considered a soft touch receptor and will be discussed further in the next section.

### 3.2. TRP Channels in Long-Lasting Touch-Sensing Cells

Long-lasting soft touches are thought to produce slowly adapting pulse trains in the nerves, resulting in sensations of comfort for humans. According to the relationship between the reversal potential and the Ca/Na permeability, Ca/Na permeability in the TRP channels of mechanoreceptors is in the middle of the range (ca. 5). Soft touch receptors may consist of Ruffini endings and Merkel cell-neurite complexes in the hairy skin, where Merkel cells release neurotransmitters to activate SA1 (slow adapting 1) afferents [28]. In this case, TRP channels of soft touch receptors would induce Ca^2+^ influx by activation of TRPC7, TRPV4, TRPM3, and TRPP, all of which have a high conductance (about 100 pS) for Ca^2+^. Ruffini endings are actually axonal terminals, in which the reversal potential is around 20 mV, meaning that the Ca/Na permeability ratio is about 5. These TRP channels are strong candidates for a role in soft touch sense receptor response [33].

### 3.3. TRP Channels in Vibration-Sensing Cells in the Glabrous Skin

In a glabrous skin, Pacinian corpuscles are deep-seated, whereas Meissner corpuscles are located nearer the surface. Both of these receptors produce simple responses to short-term stimulation and may be sensitive to strong pressure [31]. It is, therefore, reasonable to assume that TRP channels in Pacinian and Meissner corpuscles have a high reversal potential along with a high Ca/Na permeability ratio. Of the many kinds of TRP channels, only TRPV1 has been reported to be expressed in these two types of corpuscle, which have a reversal potential of about 25 mV [34]. It should be noted that TRP channels are also expressed in the glial cells of DRG and DH, where they are utilized to make chronic pain [35–37]. Evidence suggests that TRP channels in the mechanoreceptors, the DRG, the DH, and the CNS may be involved in physical analgesia [38, 39].

## 4. Pain and Analgesia in the Nociceptive System

In proposing a novel molecular mechanism for physical analgesia, we first outline the pain pathway that has been already established. The information flow from the periphery (i.e., skin, joint, and muscle) to the perception system is tissue damage to nociception to transmission to perception. From the periphery to the CNS in the above pathway, the contribution by TRP channels to transmission is restricted to the DRG and DH [36, 37]. Many signaling chemicals have been proposed including NGF, bradykinin, 5-HT, ATP, H^+^, glutamate, somatostatin, and GABA. Among them, only ATP and H^+^ are known to be associated with TRP channel activation. In the DRG and DH, essential modification of the pain signal may occur through interactions among astrocytes, microglia, and presynaptic and postsynaptic neurons.

Pain signals can be modified by an interaction between astrocytes and neurons, which are mediated through cytokines (e.g., 1L-1*β*) and chemokines (e.g., CCL2) from astrocytes to neurons and ATP from neurons to astrocytes [36]. Between microglia and neurons, cytokines (TNF-*α* and 1L-1*β*) and prostaglandin E2 are signal transmitters. Reciprocal signaling from neurons to microglia is mediated by ATP and CCL2 [37].

## 5. Novel Analgesia by Pyramidal Thorn Patch Application

In medical clinics, the mainstay of analgesia has long been and continues to be opioid therapy (see the WHO guidelines for pain: http://www.who.int/medicines/areas/quality_safety/delphi_study_pain_guidelines.pdf?ua=1). Thus, much effort has gone into decreasing opioid side effects [40, 41]. However, the efficacy of this kind of pharmacological therapy is limited. As a result, many suffer chronic tennis elbow pain, neck pain, and knee pain among others [42]. To overcome this limitation, several types of physiotherapy have been so far attempted all over the world [24]. Chiropractic, osteopathy, acupuncture, and aromatherapy have been adopted as complementary medical practices worldwide (see the NIH-NICCH website: https://nccih.nih.gov/health/integrative-health). These methods were developed in ancient times due to their effectiveness, but no molecular mechanisms of action have been elucidated. In recent years, however, a number of scientific groups, including our own, have investigated models for studying the molecular mechanisms of analgesia for chronic pain from the standpoint of physiology by modification of pain signaling in the DRG and DH from mechanoreceptors to the brain.

### 5.1. Signal Flows for Analgesia by Pyramidal Thorn Patch Application

In the following sections, we will describe another molecular model of analgesia involving the generation of oxytocin (Oxt) and nitric oxide (NO) by soft touches. Application of pyramidal thorn patches to the pain regions of the skin can reduce several types of chronic pain. Here, we will posit another hypothesis to explain how soft touches can reduce chronic pain with contributions from Oxt and NO.

Distinct from acute pain, the chronic pain signal is modified by an interaction among microglia, astrocytes, and neurons in the DRG and DH before arriving at the CNS. Pain signals from a joint angle and/or nerve injury are carried by A*α* and *β* fibers to the DRG [43]. On the other hand, touch signals produced by soft touches such as light stroking are carried by A*δ* fibers, producing an effect that is distinct from A*α* and *β* fibers on microglia and astrocytes in the DRG and CNS. Differences among these three myelinated fibers are found in fiber diameter (20, 12, and 6 *μ*m, resp.) and conduction velocity (120, 72, and 36 m/s, resp.) [44]. We should thus consider differences in interaction with glial cells among these three types of A fibers. Recently, a low threshold mechanoreceptor was suggested to be able to modulate nociceptive activity in the DRG and DH and produce inhibitory effects on spinothalamic tract cells [45]. Because the pain system engages Oxt/NO-mediated mechanisms, this finding offers support for our developing model.

**Figure 2:**
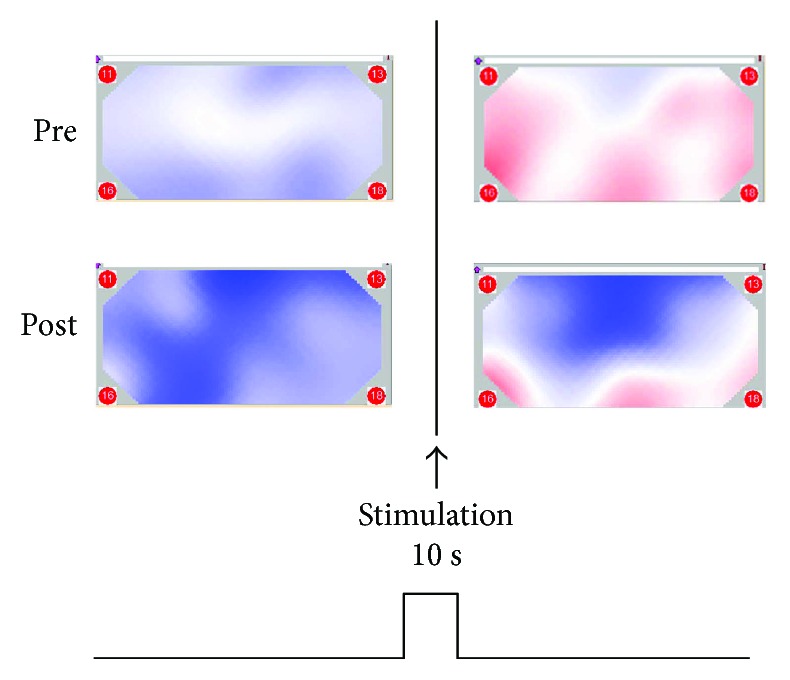
Near-infrared spectroscopic (NIRS) images of the frontal cortex of a subject with pain in the left thigh. Pre shows the images obtained from the subject to whom the pyramidal thorn patches have not yet been applied. Post shows the same subject after patches have been applied. The lower parts of the Pre images expressed in red are the frontal cortex, indicating that the concentration of oxyhemoglobin in the blood in the frontal cortex increased after maintaining a posture with pain for 10 sec. The patch application relieved the pain sensation, which can be judged in the Post image (in blue) with NIRS.

Our new model of physical analgesia may be defined as follows: soft touches to the hairy skin open mechanosensitive TRP channels of Merkel cells that allow positive ion (Ca^2+^ and Na^+^) influx to the cells. Activated cells fire SA1 (slow adapting of firing train) nerves belonging to the A*β* class, which in turn release chemicals such as Oxt and NO from their nerve endings in the DRG. The chronic pain signal is reduced to some extent in the DRG and DH and flows up to the CNS. The TRP channels in glial cells of the DRG are activated by ATP, which is released from nerve endings (glial cells probably release chemokines as well). Eventually, the chronic pain signal is reduced and then sent to the CNS through A*δ* and C fibers. The reduced pain signal is recognized in the frontal cortex of the brain [46] (Figure 2). There is a possibility that the reduced pain signal is further modified by Oxt and NO in the brain and that this is thought to cause a placebo effect, something we will discuss in more detail later.

The application of our new model of physical analgesia enables us to describe as follows. When the pyramidal thorn patches are applied to the normal hairy skin for a long duration, the Merkel cells around hair follicles are continuously activated and, too, the connecting SA1 fibers that carry the impulse trains. When these impulse trains arrive at the DRG, Oxt is released from the presynaptic endings of SA1 fibers, reducing other types of pain signal carried by A*β* (RA2). This A*β* (RA2) may also release Oxt in the DRG and DH, because this fiber can respond to any mechanical stimulus [43].

In addition to the above A*β* fiber model, the C fiber model is also possible. Emotional touches may be more psychologically effective than soft touches [15]. When pain relief is successfully achieved by C fiber firing following emotional touches, it is thought to occur via the activation of GABA A receptors of postsynaptic neurons in the DH [46]. It should be noted that the efficacy of pain relief by the activation of GABA A receptors depends on circadian rhythms. Based on the data previously reported [47], we can propose that the analgesic effect is less during daytime than at nighttime.

### 5.2. Molecular Mechanisms Involving Oxytocin in Pyramidal Thorn Patch-Induced Analgesia

Oxt is the most likely candidate molecule for chronic pain relief induced by soft touches. Oxt has an antinociceptive function [38, 48], but the molecular mechanism of pain relief has not been established yet. How does Oxt reduce the activity of pain signal-carrying nerves? Oxt is thought to hyperpolarize the pain signal-carrying nerves. Recently, Oxt was reported to inhibit not only somatic nociception but also visceral nociception [38]. Whereas Oxt cannot pass through the blood-brain barrier, a nasal application allows it to reach the brain [49]. Thus, a reasonable explanation for an antinociceptive function of Oxt is that it can reduce pain signals in the DRG [38, 50]. The analgesic effect of Oxt, therefore, appears to occur by hyperpolarization of neurons in the DRG.

**Figure 3:**
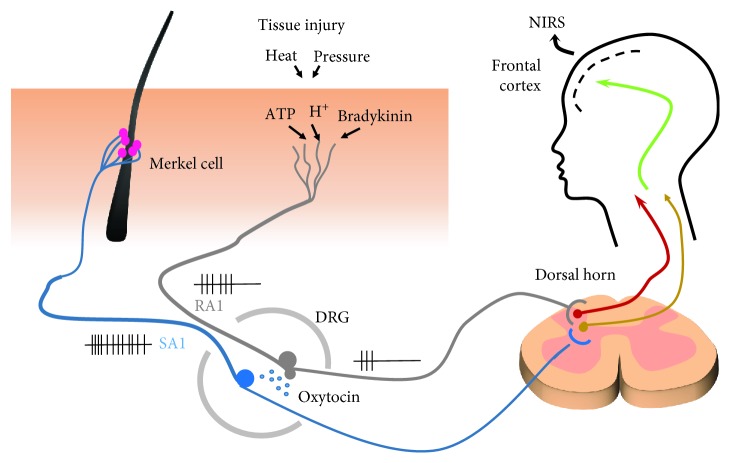
Proposal mechanism for physical analgesia by soft touch involving oxytocin in the DRG. As shown in Figure 2, the pain sensation can be recorded at the frontal cortex by near-infrared spectroscopy (NIRS).

How does Oxt hyperpolarize DRG neurons? Oxt receptors belong to the G protein-coupled receptor family [50, 51], and several types of TRP channels are expressed on the surface of DRG neurons [52, 53]. Activation of Oxt receptors induces inositol trisphosphate (IP3) release to the cytosol and diacylglycerol (DG) production in the membrane of neurons. IP3-induced Ca^2+^ and residual DG in the cell membrane can activate TRPC channels, which regulate Ca^2+^ influx [50]. Increased intracellular Ca^2+^ then activates Ca^2+^- and voltage-activated K^+^ channels (BK (Ca)), leading to K^+^ efflux and DRG neuron membrane hyperpolarization. Thus, the initial pain signals carried by rapid adaptation (RA) type nerves are reduced. Importantly, in this model, pain signals are carried by A*δ* (RA1 and 2) and C fibers, and soft touch signals are carried exclusively by A*α* (SA1) fibers (Figure 3).

## 6. Perspectives

TRP channels and Oxt, the main mechanosensitive channel and chemical mediator in pain relief, are the key elements of this review [38, 54, 55]. Activation of Oxt receptors activates TRP channels in the vicinity of Oxt receptors intracellularly, inducing the hyperpolarization of the DRG neurons. According to our proposed models, alcohol and heat (i.e., TRP channel activators) can be used to relieve pain. In support of our model, Salat et al. proposed that an agonist of TRPV channels may be a novel drug for pain treatment [56]. NO-activated TRPA1 channels are another candidate target for pain relief [57]. In addition, we propose that TRPC channels are involved in analgesia, because TRPC7(−/−) mice have no pain sensitivity (unpublished data). In addition, DG formed from the decomposition of phosphatidylinositol 4,5-bisphosphate (PIP2) by phospholipase C (PLC) may activate TRPC channels in besides the activation of protein kinase C (PKC) in the typical signaling pathway.

Intracellular signaling following activation of Oxt receptors still constitutes an unresolved pathway. Pain-sensitive DRG neurons are hyperpolarized by Oxt via a Ca^2+^/neuronal nitric oxide synthase (nNOS)/NO/ATP-activated K^+^ channel pathway [58]. Increased intracellular Ca^2+^ changes membrane potential by reducing intracellular K^+^ concentration. If a change in the membrane potential appears within seconds, our model is likely accurate. If it takes more than 30 minutes, we have to consider the contribution of a phosphorylation effect by Ca^2+^-dependent protein kinases (e.g., Ca^2+^/calmodulin-dependent protein kinases (CaMK) II and IV and protein kinase C (PKC)) in the process, in which case the hyperpolarized state would continue for a long time (days).

The next area of significance is placebo analgesia and mindfulness meditation-mediated pain relief [59, 60]. Both methods of analgesia function upon the brain but are independent of the periphery. Their respective mediating molecules may be Oxt and NO, which are associated with nerve activation in the brain, specifically hyperpolarization of pain-related nerves, and thus a type of action quite similar to that of opioid [38, 61].

Finally, we propose that several TRP channels are the final targets of analgesia mediated by Oxt and NO. These TRP channels induce analgesia in the brain and the DRG and DH by hyperpolarization of pain-related nerve fibers [62]. It remains to be understood, however, how both Oxt and NO serve as the key molecules of physical analgesia and, furthermore, why TRP channels can regulate longevity and metabolism [63]. Whereas the gate theory, which is similar to our model, has been previously proposed for analgesia with due regard to the function of only DH [64, 65], our models proposed in this review including the function of the DRG in addition to the DH should be distinguished as addressing the molecular mechanism underlying physical analgesia for chronic pain. We would like to propose our model as an “innovative gate control theory.”

## Figures and Tables

**Table 1:** Selective combination of mechanoreceptors and nerves.

Skin	Receptor	Frequency	Nerve	Modality
Glabrous skin	Meissner corpuscle	1–300 Hz	A*β* (RA1-LTMR)	Stroking
				Fluttering
	Merkel cell	0–100 Hz	A*β* (SA1-LTMR)	Pressure
				Texture
	Free nerve ending	0–8 Hz	—	—
	Pacinian corpuscle	5–1000 Hz	A*β* (RA2-LTMR)	Vibration

Hairy skin	Merkel cell		A*β* (SA1-LTMR)	
	Hair flattening		A*δ* (SA1-LTMR)	

RA: rapid adaptation; SA: slow adaptation; LTMR: low-threshold mechanoreceptor.
